# Burnout at Work in Modern Times

**DOI:** 10.14740/jocmr2281w

**Published:** 2015-08-23

**Authors:** Sofia Neves Pinheiro da Costa, Luis Henrique Amorim Teixeira, Luiza Neves Pinheiro Bezerra

**Affiliations:** a Department of Neurology, Restauracao Hospital, Recife, PE, Brazil; b Department of Psychiatry, Medical Institute Teacher Antonio Figueira, Recife, PE, Brazil; c Department of Psychiatry, SESAU, Recife, PE, Brazil

**Keywords:** Burnout, Work, Psychiatry

## Abstract

The theme of this research is burnout at work in modern times. The main objective is to analyze aspects of mental health worker. The specific objectives are to evaluate the issue of health and mental illness in the workplace, to understand the field of psychodynamics of work, and to analyze the work and the mental strain. The methodology used is the literature review. We conclude that is not the hostile environment that directly causes burnout and other conditions, but the inability to deal with the powerlessness of the working conditions.

## Introduction

This study investigated the causal conditions and manifestations of the burnout syndrome at the workplace. Thus, it is possible to recognize the genesis of a public health issue - burnout at work - that can lead to depression and psychological malaise.

Most legislations recognize stress and depression as occupational illnesses. However, it is necessary to prevent burnouts and suffering at work, before they become a pathology, which can be considered a generic problem with serious implications both at work and beyond.

Workers’ burnout, when analyzed based on the psychodynamics of work, contributes to the understanding of psychological mechanisms where their work is a structural element. However, the sharing of professional values can engender sublimation as the primary factor ensuring pleasure at work, but this merely occurs through a collective construct that is guided by the predominant values of their backgrounds.

Teamwork for encountering challenges and possibilities in everyday situations will enable workers to surpass the differences in their backgrounds, resolve work conflicts, and overcome intra-psychological conflicts, thus leaving an open path for either resilience or depression.

Thus, the main objective of this study was to analyze aspects of workers’ mental health. The specific aims were to evaluate the issues of mental health and mental illness at work, to understand the psychodynamics of work, and to analyze work and burnout. The methodology used is literature review, through theoretical research of books and journals related to the theme.

## Mental Health at Work

The feature of “work” has been extensively studied due to its relative effects on the mental health/illness of workers. This theme, under various circumstances, has generated research studies which focus on the inception of the varieties of burnout among workers from the various fields of production.

The association of the health/illness processes begins to be studied in its determination of the work life, by the enrichment of the analytical axis and by establishing a perspective in which the purposes of the investigations assume ethical directives [1].

Thus, the fundamental principles of the studies superseded the pursuit for productivity and engaged in identifying aspects that might cause burnout, “even if they are simultaneously in the service of production interests” [1].

According to Seligmann-Silva [1], this evolving feature of research, that is, work and mental health, is primarily based on interdisciplinarity and attempts to integrate distinct views of the biological, human, and exact sciences. It seeks to analyze the association between working and health/illness, the dynamics of which are primarily apparent on mental phenomena, even if their nature is eminently social.

Some subjects such as work medicine, work psychology, psychopathology of work, toxicology, and ergonomics have studied mental processes and/or health/illness dynamics of human beings when they are subjected to different working conditions. These subjects are based on physiology, neurology, psychiatry and psychosomatic medicine, and psychoanalysis [2].

For Ramazzini [3], other subjects study human work directly or indirectly not assuming health to be their formal object, as is the case with political economy. This characteristic is also observed in every subject that converges the work organization in its capitalist feature, as is the case of sociology and business management, which focus on company and production.

The theoretical support for the psychodynamics of work offers a better grasp of the causal conditions and manifestations of burnout in the course of work, for it allows the focus to be on “the conflicts that arise from the encounter of a pre-existing singular subject and a work situation the characteristics of which are largely fixed, regardless of the subject’s will” [4].

This implies that we have a previously constituted subjectivity that will be later exposed to the reality of work, which may occasion a transformation in our conducts when we are facing a conflict, or a transformation in the work reality may also occur through a supplementation of our subjectivities [1].

In the psychodynamics of work, mental illnesses are not the focus of analysis, rather the behaviors and attitudes that indicate mental suffering and the struggle against mental illness are concentrated upon. One’s mental life is not “reduced to (objective) factors in the same way that a machine is analyzed in terms of its components and parts”; the subjective experiences of the workers should be considered [1].

According to Dejours and Abdoucheli [5], mental illness only makes sense for a particular individual when it is registered in the private domain. Work, with its social nature, absorbs workers from the social environment, registering itself in the collective domain. Thus, when encountering identical work pressures, the consequences on mental functioning are not the same, for there is a private-social conflict, where it is observed that the private vigorously resists.

For Codo and Jacques [6], between work pressures and mental illness, there is the individual, who is capable of understanding their situation, reacting and defending themselves. In the psychodynamics of work, the conflict beyond the personality domain or its singular reactions is what is pursued, that is, in the concrete reality of organizational logic. In that way, workers build private defense reactions considering three functional variables: their personal history, the structure of their personality, and the work organization.

Considering that the psychodynamics of work constitute only a form of thinking for this study’s broad objective, other theoretical perspectives have been integrated to escape reductionism. This is a contemporary tendency and may be verified in the recent studies on work and mental health.

This is why the concept of burnout was chosen, based on the theoretical construct of Seligmann-Silva [1] and not on psychological suffering, as developed by Dejours [7].

## Psychodynamics of Work

Considered an evolution of the psychopathology of work, and therefore, also called the new psychopathology of work, this study is more oriented toward identifying specific mental illnesses correlated to the profession or to the work situations. Its field of investigation explores the participants who, despite the work pressures, are able to avoid illness and madness. Therefore, it studies the aspect that is unaffected by the mental pressures.

The psychodynamics of work first appeared in the studies by Christophe Dejours during the late 1980s in France, having a strong impact on the views presented by the psychopathology of work. Its followers founded the Dejourian School, gathering various specialists and research fields.

Dejours [5] proposed a new appreciation of the concept of work-related mental load, which cannot be quantified, for it is qualitative and as an experience cannot be measured, such as pleasure, satisfaction, frustration, aggressiveness. The concept of psychological load is related to the affective and relational aspects of work, and it is an economical approach to psychological functioning. According to Dejours and Abdoucheli [5], “as for psychological load, the main danger is of an underemployment of psychological aptitudes, either phantasmic or psychomotor, which cause a retention of drive that precisely constitutes the psychological load of work.”

In that way, if the workers are not able to find a freely chosen or organized job, they will have their ways of unloading poorly adapted to their needs. Psychological energy will accumulate, becoming a source of tension and displeasure. In this case, the psychological load will be positive, otherwise, the psychological load will be negative, and the work will be considered as a balancing agent.

In the psychodynamics of work approach, the primary concern is with the most comprehensive dynamics regarding inception and transformations of mental suffering linked to the work organization [1].

“It is a treatment of men at work” [4]. Through this view, the notion of suffering preliminarily implies a state of struggle of the participant against forces that are inclined toward mental illness, and those forces should be identified in the work organization [8].

The first analysis of suffering designates the field that separates illness from health. In a second analysis, suffering arises when the negotiation between a man’s liberty and the prescribed work organization reaches its ultimate limit, and the man-work relationship breaks down [1].

According to Ferreira [8], work organization is not only: “division of work, that is, division of tasks among operators, rhythms imposed, and operational modes prescribed but also, and above all, division of men to guarantee this division of tasks, represented by hierarchies, sharing of responsibility, and control systems.”

Pathogenic suffering occurs when the work organization is in conflict with the psychological functioning of men and all the possibilities of adaptation between the work organization and desire collapse. Then, as a form of mediation, for all this is a dynamic process, individuals create forms to protect themselves, developing defensive strategies.

The conflict that opposes a worker’s desire and the reality of work creates a confrontation between the worker’s spontaneous project and the work organization, which limits the performance of that project and dictates a precise mode of execution [4].

According to Dejours [9], “work can cause unhappiness, alienation, and mental illness as much as it can be the mediator for self-realization, sublimation, and health”. Therefore, in the psychodynamics of work, mental suffering is conceived through both aspects, that is, it can lead to either illness or creativity.

The challenge in this field of study is to overcome the present gap between the prescribed work organization and the real work organization considering the dangers that this distance “represents health, safety, and quality of what is produced” [1].

In this sense, Pitta [10] states that: “the distance between prescribed work and real work, [ ]is a demonstration that, without certain arrangements individually developed by each worker, the work prescriptions and routines produced in [ ]any space of standardization are hardly executed as they are prescribed, and once this happens, nothing guarantees that results will be those expected.”

In the psychodynamics of work, the socio-historic perspective and the work organization are considered, using the psychoanalytical reference integrated into a dialectic vision.

In those studies, psychoanalytical concepts have served as a support to their theoretical construct for the purpose of analyzing mental suffering associated with work and the genesis of that suffering. The notions of mental suffering and pleasure associated with work, used in psychodynamics of work, are related to anguish and desire, both studied in psychoanalysis.

In the development of psychodynamics of work, Dejours [7] also applies the concepts formulated by Pierre Marty that investigate psychosomatic economy, also anchored on the psychoanalytical reference.

Through that approach, the correlation of processes and work-conflicts can often be established by psychosomatic disorders. Seligmann-Silva [11] explains that Marty’s theory is based on the concept of psychosomatic economy, where “under certain circumstances, a progressive disorganization can be seen in the established psychosomatic functioning, followed by a reorganization in which there is a return to the functioning that existed in phases prior to the development of the individual.”

This is exemplified through the massive expression of emotions by visceral functional modifications, such as an increase in intestinal peristalsis “in situations of fear, instead of using the psychological processing typical of a more advanced and improved stage of the psychosomatic system organization” [11].

However, Dejours [7] exemplifies how people present different levels of psychosensory demands, such as the need for varied work, according to their personality needs. In this sense, he says: “the determinant role of psychiatric decompensation [ ]was played by the neutralization of behavioral defenses during a change of work position [ ] “an improvement” of work conditions, with a reduction of physical load, can lead to a catastrophe at the level of the individual’s general economy with their pathological clinical translations, if applied blindly, without considering the personality needs.”

Thus, some people need strong psychosensory demands. The more changes, varied sounds, loud music, less monotony, and routine, the better they feel. When they are deprived of that, they become unbalanced, developing a somatic disease.

Based on the studies by Seligmann-Silva, it is verified that human variations do not indicate a rigid standardization, in movement as well as in sensory and mental activities that compose the work performances, whenever protecting or promoting workers’ health is considered [11].

These factors constitute work as a balancing agent to the structure of a personality. In this situation, the psychological load is negative, it is part of the pleasure at work and it counterbalances, in part, physical and nervous loads, to the point that it assures these workers a balance. In other words, according to Dejours [7], “what is important is to understand the simultaneity of pleasure and necessity.”

## Work and Burnout

Work organization precedes an entire analysis on the question of the workers’ quality of life, for in it, they are submitted to physical and psychological pressures: 1) by the direct action of work load; 2) by working environment conditions; 3) by insufficient salaries for a dignified life [9].

Currently, in addition to profit, which is the primary objective of a company, what characterizes it is the profile adopted by the organization, planning, and management. Their concern is not as much related to the promotion of direction and planning, for they have always occupied an important place. According to Ferreira [8], the current concern is to demote work, the “centrality” of which is now being contested on the economic level as well as on the social and psychological levels.”For the author, this situation constitutes the “nucleus” of neoliberalism in work environments.

According to the economicism prevalent in the analyses of neoliberal theses, the future belongs to the industries. This idea seeks to deny the centrality of work, even if in the current socio-economic context people still work more, the real duration of work increases, and the work force is increasingly outsourced, among other things.

Work here is understood as a general and homogeneous reality, a social relationship, a mode of using the work force. The statute or role in which are gathered the particular function fulfilled by the individual in the company, the form of his task, the qualification that is acknowledged in him, and the privileges that are given or refused to him are empirical aggregates, resulting from the social functioning of the isolated company. These are notions that cannot be confused with the notion of work in the broad sense [12].

According to Ferreira [8], the system of relationships that consequently exist in a company, in essence, is not within the workplace, but determines it. The meaning of work is much more than the mere execution of tasks in the system of relationships within the company, but it permeates the ambit of family and society. This is the meaning that is obliterated by neoliberalism.

In this way, the work organization, mediating the system of relationships between social actors, possesses a centrality in the determination of the causal conditions and manifestations of burnout, reaching its psychological dimension.

For Dejours and Abdoucheli [5], the work organization is conceptualized “by the contrast in the working conditions, on which most medical and ergonomic researchers focus their studies.”

According to these authors, the work organization is the division of tasks among the team, the imposed rhythms, the operational modes demanded to execute the labor activities, and the division between its participants to guarantee the sharing of tasks and responsibilities, which is represented by the managers, through the control systems they exercise. It is a socially constructed relationship.

The work organization includes the “division of work: division of tasks among the operators, sharing, rhythm, and finally, the prescribed operational mode; and the division of men, division of responsibilities, hierarchy, command, control, etc.” [5].

Leopardi [13], referring to the questions related to work, says that in the conceptions on its development viewed from the political-economic perspective, people are perceived as mere objects, whose value depends on the variation of a certain currency in the payment to a work force.

Seligmann-Silva [11] supplements these concepts, stating that the interests of capital in the work organization function to guarantee, simultaneously, “the maximum efficacy in the production process, the lowest cost relative to work, and the maximum possible subjection of paid workers.”

However, it is necessary to rethink work in reference to the “notion of live subjects relating” [13], a direction that approximates the proposal by Bourdieu [14] that “the real is relational,” and thus, it may happen that I know nothing about the work that I claim to know all about, because it is nothing outside of its relationship with the whole. In this way, we can perceive the current conformity of work organization as a social relationship in our world.

Thus, for Jackson Filho [15], the hierarchy, and the division of tasks and people are considered essential initial factors to understand the work organization. In these factors, there is a fundamental articulation between the work process, the form of administrating this process, and mental health. Pressures within this articulation are derived from the work organization.

Furthermore, the pressures presented by work conditions such as physical, mechanical, chemical, and biological pressures of the work position on the worker’s body may create an imbalance on the psychological system, causing burnout.

According to Pires [16], the psychological load of work functions as a “regulator of the worker’s psychological system.” If the work allows this unloading, it functions as a balancing instrument for the worker, and the work can be a source of pleasure, through the relief of that psychological load at work.

To the extent that the work organization is the will of another person, that is, of someone who holds the power in a given institution, it counterpoints the worker’s desire, limiting his participation in this process by establishing the execution process in a trim and precise form from outside. ( ) workers must act according to the will of their hierarchical superiors, and their free will is replaced by the imposition of managers. The psychological load is, therefore, the result of a confrontation between desire and the orders of their superiors [4].

In this sense, Marx [17] underscores that the work becomes something external to the worker, not belonging to his nature and not constituting the satisfaction of a need: “The exteriority of work for the worker is shown in the fact that it is not his own work, but another’s, in the fact that it does not belong to him, that while at work he does not belong to himself, but to another.”

## Conclusion

This study presented the theoretical fundamentals involving the issue of burnout at work. In the psychodynamics of work of the Dejourian School, it was possible to apprehend the object of the study through the conflicts evident in work situations, making it possible to understand the work organization as an intrinsic factor in the constitution of the burnout syndrome.

The theoretical construct of Seligmann-Silva enabled the use of the concept of burnout associated with the sense of loss, thus expanding its conception. In this sense, every loss engenders burnout. Since it does not affect productivity, burnout is not valued, for after all, individuals continue to work, even under precarious working conditions.

The conditions that generate burnout at work frequently appear; hence there is a confrontation between the will and desire of workers and the demands from the organization’s command.

Considering all the conditions for workers to perform their activities, the burnout experienced at work has weakened individuals. The authors’ research has indicated that these professionals cannot process their work experiences. Their capacity for affective mobilization is dulled, thus they cannot overcome the perception of impotence in face of the conflicts related to the work organization.
